# Dermatofibrosarcoma protuberance in a black African cohort—a clinicopathologic study

**DOI:** 10.3332/ecancer.2020.1086

**Published:** 2020-08-07

**Authors:** Gabriel Olabiyi Ogun, Uchenna Simon Ezenkwa, Omobolaji Oladayo Ayandipo

**Affiliations:** 1 Department of Pathology, University of Ibadan/University College Hospital, Ibadan, 200001, Nigeria; 2 Department of Surgery (Division of Oncology), University of Ibadan/University College Hospital, Ibadan, 200001, Nigeria; a https://orcid.org/0000-0001-8535-3240

**Keywords:** dermatofibrosarcoma protuberance, DFSP, sarcoma, Black Africans, clinicopathologic, Nigeria

## Abstract

**Background:**

Dermatofibrosarcoma protuberance (DFSP) is the commonest, yet rare, dermal sarcoma globally. There are few reports in the literature of this neoplasm in Nigerians and indeed in sub-Saharan Africa. This study documents our institutional practice observation and compares it with those from other regions of the world.

**Methods and materials:**

This study was a retrospective review of all cases of histologically diagnosed DFSP at the University College Hospital, Ibadan, Nigeria, spanning a period of 27 years (January 1989–December 2016). Data on patient age, gender, tumour location, size, tumour recurrence and metastasis status were obtained from clinical and surgical pathology archival files and records.

**Results:**

Sixty-nine cases of DFSP were recorded over the period reviewed with a male–female ratio of 1.6:1. The mean age of the study population was 39.6 years. The youngest patient was 5-year old, while the oldest was 86 years and the modal age group was the 4th decade. The trunk was the commonest anatomic tumour location. Recurrences were seen in seven cases with recurrence interval ranging from 6 to 240 months. The correlation between tumour size and age was non-significant (*r* = −0.183; *p* = 0.182). There was fibrosarcoma-like transformation in three cases (4.3%) studied.

**Conclusion:**

Dermatofibrosarcoma protuberance is rare in our population and occurs more commonly in males and on the trunk. Recurrence can occur beyond the recommended follow-up period of 10 years.

## Introduction

Dermatofibrosarcoma protuberance is the most common dermal sarcoma of unresolved histogenesis and seen more frequently among middle-aged individuals [1, 2]. It is a rare neoplasm with an incidence of about 4.1 per million person years among the American population [3]. Studies have shown a higher occurrence among the white population in multiracial studies [3, 4]. Gender predilection is variable [3–5] with some studies reporting equal sex occurrence [6, 7].

It is known that DFSP can occur at any location in the skin. Different studies show differences with regards to the commonest site of occurrence [3–5, 8]. The occurrence in unusual locations such as the acral sites [9] and the perineum/vulva [6] have also been documented. This neoplasm has an indolent growth with frequent recurrences owing to its invasion into subcutaneous tissue. It, however, rarely metastasizes. It is, therefore, regarded as a low-grade sarcoma with excellent 10 year survival of 99% [2, 6]. Studies on dermatofibrosarcoma protuberance from Nigeria are few [7], hence the need to undertake this study. More so, cases of missed diagnosis are frequent with patients often presenting as a recurrence before accurate diagnosis is made [8, 10]. Thus, this study reviewed the clinicopathological features of cases of dermatofibrosarcoma protuberance seen in our hospital and compared these to studies in other populations.

## Methods

We retrospectively reviewed all the cases of histologically diagnosed dermatofibrosarcoma protuberance at the University College Hospital (UCH), Ibadan, Nigeria spanning a period of 27 years (January 1989–December 2016). Data on patient age, gender, tumour location and size, tumour recurrence, transformation with fibrosarcoma-like features and metastasis status, if any, were obtained from records archived at the Department of Pathology, UCH, Ibadan. Haematoxylin and eosin slides of all the cases were in addition retrieved and reviewed to reconfirm diagnosis of dermatofibrosarcoma protuberance. The cluster of differentiation (CD) CD34 was applied to support the diagnosis in some of the cases. All data were anonymised and handled according to the institutional guidelines. Descriptive statistics was used to determine frequencies, mean and median of the variables as indicated. Pearson correlation statistics was used to determine the relationship between patient age at first diagnosis and tumour size. Patient age was dichotomised using age 40 as a cut-off (this was the mean age of the study population); while the tumour size was dichotomised using tumour size 5 cm as a cut off. All statistical analysis was conducted using SPSS version 20 (IBM Corp, 2011). Outputs were presented as prose, charts and tables. This study was conducted in compliance with the guidelines of the Helsinki declaration on biomedical research in human subjects. Confidentiality of the identity of the patients and personal health information was maintained

## Results

**Figure 1.**
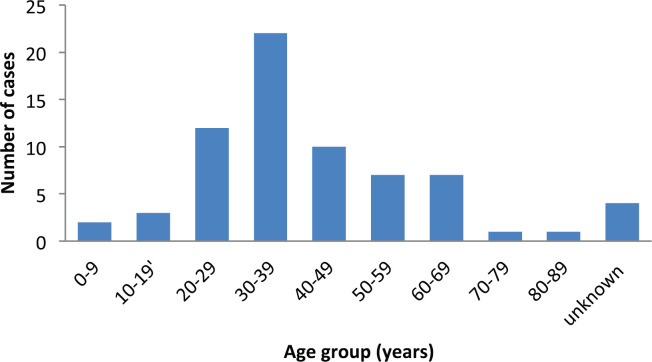
Number of patients per each decade of life.

**Figure 2.**
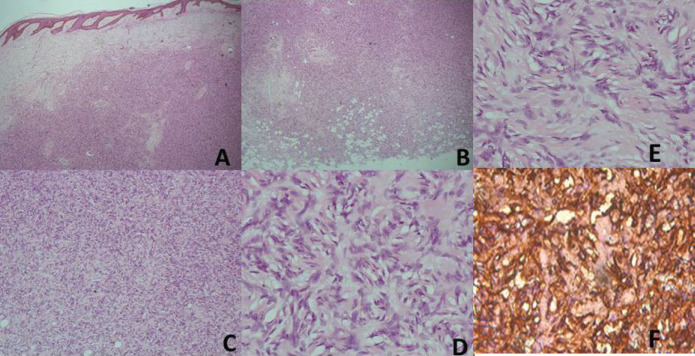
(A) Haematoxylin and Eosin (H&E) x40; Low power view with the tumour in the dermis. (B) (H&E), ×40; The deep margin of resection with tumour infiltrating adipocytes. (C) (H&E), ×100; Prominence of the marked cellularity of the tumour. (D and E) (H&E), ×400; Typical morphology with cartwheel arrangement of the spindle tumour cells with collagenous stroma more prominent in E. (F) (CD34), ×400; Strong staining of the spindle cells with the collagenous stroma unstained and prominent vascular channels highlighted by CD34.

Sixty-nine cases of dermatofibrosarcoma protuberance were recorded over the period reviewed. This comprised 42 males (60%) and 26 females (37.7%) with gender of one case not documented. Male/female ratio is, therefore, 1.6:1. The mean age of the study population was 39.6 years. The youngest patient was 5-year old, while the oldest was 86 years. The modal age group was the 4th decade (Figure 1) with most (62.3%) of the patients being 40 years or younger (Table 1). Tumour size ranged from 1 to 28 cm and the median tumour size was 8 cm. Anatomic locations of the tumours included the head and neck, upper extremity, trunk, perineum and lower extremities as shown in Table 1. The typical histological features (Figure 2) were those of storiform (‘cartwheel’) arrangement of relatively monotonous spindle cells with elongated nuclei, minimal cytologic atypia and scanty eosinophilic fibrillary cytoplasm within a collagenous stroma and typically infiltrating subcutaneous adipocytes with strongly CD 34 staining which is typical for DFSP (Figure 2F). In seven (10%) of the cases, CD34 staining was performed on Formalin fixed paraffin embedded (FFPE) tumour tissue. There were seven cases with recurrence, five of these presented with recurrence at the time of first presentation to our hospital, the patients could not accurately state interval of recurrence, whilst the remaining two cases were seen in one patient who had recurrences at 6 months and 11 months following resections in our centre. Two of the primary recurrences occurred 44 months and 240 months following the first excision, the former being in the scalp while the latter was in the trunk. The other three cases of recurrences had no documented recurrence interval; two of these were on the buttocks and one was located on the anterior abdominal wall. Surgery was the primary mode of treatment in all the patients in our cohort, recurrence was likely due to surgical resection margins not been free at the initial surgical excisions because DFSP is usually not thought of as the initial clinical diagnosis. Correlation between tumour size and age was also non-significant (*r* = −0.183; *p* = 0.182). Three cases had fibrosarcoma-like transformation with prominent fascicles and with all having mitosis of greater than 15 per 10 high power field and with increasing atypia of the tumour cells and areas of necrosis noted.

## Discussion

The rarity of DFSP is shown by the small number of cases reported in various studies from different parts of the world, some of which span more than two decades [6, 7, 10]. This current study is another report in that regard. This current study, like those studies, was hospital based and differs slightly from the population studies in the United States [3, 4]. Despite this, Kreicher *et al* [3] showed that the incidence of this neoplasm is rather low when compared to the large population from where they were drawn. Given that these tumours are indolent and rarely metastasize, making an effort to understand its precursor lesion may help to reduce its incidence even further.

Some of the findings in the available literature have been shown in our study. These include wider age of occurrence, predilection for the trunk, few recurrences and, in our study, male gender bias.

Although DFSP is said to be seen more in middle-aged adults,[11] recent literature suggests a higher frequency among the younger population [3] as also shown in the present study, however that observation might be due to the relatively young population structure in Nigeria. The occurrence in individuals 5-year old or younger has been reported [7, 10] with one being in south-west Nigeria [7]. Our study supports these findings with the majority being younger than 40 years, it also supports the finding by Kreicher *et al* [3] in the United States which reported modal age of occurrence at 20 to 39 years. Most studies report a predilection for truncal location as has been shown in this study [3, 4, 6, 8], while a study in Italy and another in Thailand reported the most common tumour site as the extremities [5, 10]. Occurrence at other sites occur including perineal and acral sites are varied in incidence according to different studies [6, 8–10]. This diverse tumour locations may suggest multiplicity in risk factors for these tumours as has been shown by molecular profiling [2].Tumour recurrence after complete surgical excision with negative margins is common with a study reporting as high as 22% [6]. This is higher than the 10% reported in our study and 1.5% reported by Stacchiotti *et al* [5] in Italy. In our study, however, most cases presented with recurrence at the time of first consultation in our hospital, hence, negative tumour margins could not be ascertained. This present study has shown a recurrence following 20 years after primary tumour resection and this may be one of the longest duration so far reported in literature with a few reporting 10 years [8], while cases showing recurrence few weeks after resection has also been documented [8]. Patients could therefore be expected to develop recurrence beyond the recommended follow up interval [6].We believe that this study is one of the few to report male predominance [5] compared to most studies reporting more cases in females [3,4,8,10], with a study in Nigeria showing equal gender incidence [7]. Another study that reported male predominance considered only acral site tumours, hence, the study may not be representative of all cases seen in that population. Indeed, a subset of DFSP tumours have shown progesterone receptor positivity [8], suggesting a role of gender in its tumourigenesis. While male gender has been associated with poorer survival [3, 4], role of gender have not been demonstrated in these tumours. Sarcoma transformation was observed in only 4.3% (three cases) of our cohort which in lower than 10%–15% documented in the literature [10]. We could not ascertain for sure if any of these had metastasis because of the retrospective nature of the study. The sample population in this study lacks enough power for predictive studies. Likewise, lack of comprehensive follow-up data made it difficult to undertake survival statistics. Despite these limitations, apart from the population being a black race, other known poorer prognostic factors have been documented in the present study are a predominant male gender and large tumour size [[2, 3, 6].

## Conclusions

Dermatofibrosarcoma protuberance in this study population is commoner in males and on the trunk. Although cases of recurrence are few, our finding has identified a recurrence interval well beyond the recommended follow-up interval of 10 years. We believe that this might be one of the longest recurrence intervals in recent literature. More studies are needed to present more comprehensive data regarding this tumour in our population.

## Conflicts of interest

None declared by the authors.

## Source of funding

None.

## Authors’ contributions

GOO initiated the study. GOO and USE retrieved the cases, reviewed the histology and analysed the data. OOA was the surgeon who operated on some of the patients and provided the clinical profile of patients. GOO, USE and OOA wrote and approved the final draft of the manuscript.

## Figures and Tables

**Table 1.** Patients and tumour characteristics.

Variable	Frequency	Percentage
**Gender** Male Female Not stated	42 26 1	60 37.7 2.3
**Age (years)** ≤40 >40 Not stated	43 22 4	62.3 31.9 5.8
**Tumour location** Head and Neck Lower extremity Perineum Trunk Upper extremity Not Stated	10 16 3 25 5 10	14.5 23.2 4.3 36.3 7.3 14.5
**Tumour size** ≤5cm >5cm Not stated	18 39 12	26.1 56.5 17.4
**Recurrence** Yes No	7 62	10.1 89.9
**Tumour recurrence site** Head and neck (Scalp) Trunk Lower extremity (Buttock) Not stated	1 2 2 2	
